# Supplementary material for: Should We Scale-Up? A Mixed Methods Process Evaluation of an Intervention Targeting Sedentary Office Workers Using the RE-AIM QuEST Framework
Source: Int J Environ Res Public Health. 2019 Dec 29;17(1):239. doi: 10.3390/ijerph17010239 (PMC6981814; doi:10.3390/ijerph17010239)
Supplement: Supplementary file 1 [file ijerph-17-00239-s001.pdf]

# Supplementary file 1

Non-participants will receive one of three emails (depending on their grouping – either did not participate in survey, did not participate in monitoring, or did not participate in intervention). See Appendix A. This single question will be embedded in the email via Qualtrix.

For participants who completed all phases of the study, a series of questions will be distributed to them via Qualtrix when they come for their second fitting of the ActivPal. See Appendix B.

## APPENDIX A

Email to participants who did not participate in the SURVEY.

At the beginning of the Fall 2016 semester you received an invitation to participate in a campus wide survey about your activity patterns during your work day. Not all members of the campus community chose to answer this survey. In order to try to reach all parts of the community in the future we are conducting a follow-up survey to assess reasons for non-participation. If you are willing to give your feedback, please answer the following question. This survey is anonymous.

I did not participate in the survey because:

- I did not receive the survey
- I was too busy
- I forgot to respond
- I'm not interested
- I thought it would take too long
- I feel as though this information (how I spend time at work) could be used against us
- Other (please specify) ________________________

Please add here any relevant detail to explain your answer:

| |
|---|
| |

Email to participants who answered the survey but did not participate in the study.

At the beginning of the Fall 2016 semester you participated in a campus wide survey about your activity patterns during your work day. At the end of the survey you were offered the opportunity to participate in a follow-up study by wearing an accelerometer. In order to improve future outreach efforts, we are conducting a follow-up survey to assess reasons for non-participation. It would be very helpful for us to know why you chose not to participate in the follow-up study. If you are willing to give your feedback, please answer the following question. This survey is anonymous.

I did not participate in the accelerometer study because:

- I did not understand what it would entail
- I'm not interested in the information
- I was too busy

- I forgot to respond
- It was not convenient for me
- I felt uncomfortable with having the device attached to my body
- I was worried about a skin reaction to the device
- I feel awkward or embarrassed about coming to the lab
- I already stand/move a lot in my occupation
- I feel as though this information (how I spend time at work) could be used against us
- Other (please specify) ________________________

Please add here any relevant detail to explain your answer:

<u>Email to participants who did not participate in the INTERVENTION</u>.

In the Fall 2016 semester you participated in a study about your activity patterns during your work day. Following this you were offered an opportunity to receive a personalized intervention to go over your results and discuss strategies to potentially incorporate more movement into your working day. In order to make future programs more accessible to all members of the campus community, to would be helpful for us to know why you chose not to participate in the follow-up intervention. If you are willing to give your feedback, please answer the following question. This survey is anonymous.

I did not participate in the intervention because:

- I was not interested in the information
- I was too busy
- I forgot to respond
- It was not convenient for me
- I already stand/move a lot in my occupation
- I feel as though this information (how I spend time at work) could be used against us
- Other (please specify) ________________________

Please add here any relevant detail to explain your answer:

# APPENDIX B

## Questions for participants who completed the intervention

1. How would you rate your overall health?

Excellent Good Fair Poor Very poor

2. Did your awareness of time spent sitting change following the intervention?

A lot A fair amount Moderately A little Not at all

3. Did you change your work activity patterns following the intervention?

A lot A fair amount Moderately A little Not at all

4. Overall, how would you rate the "Sit Less" intervention?

Excellent Good Fair Poor Very poor

5. How helpful was the one-on-one or small group meeting?

Very helpful Fairly Helpful Somewhat helpful Minimally helpful Not at all helpful

6. How informative was the one-on-one or small group meeting?

Very informative Fairly informative Somewhat informative Minimally informative Not at all

7. How useful were the weekly emails?

Very useful Fairly useful Somewhat useful Minimally useful Not at all useful

8. Would you change anything about the intervention?
   a. Yes
   b. No

   If yes, please explain (open) ________________________________________

9. Any other comments or suggestions? (open) ________________________________
